# Implementing a Dutch Physical Therapy Intervention Into a U.S. Health System: Selecting Strategies Using Implementation Mapping

**DOI:** 10.3389/fpubh.2022.908484

**Published:** 2022-07-11

**Authors:** Anne Thackeray, Jackie Waring, Thomas J. Hoogeboom, Maria W. G. Nijhuis-van Der Sanden, Rachel Hess, Julie M. Fritz, Molly B. Conroy, Maria E. Fernandez

**Affiliations:** ^1^Department of Physical Therapy and Athletic Training, University of Utah, Salt Lake City, UT, United States; ^2^IQ Healthcare, Radboud Institute for Health Sciences, Radboud University Medical Center, Nijmegen, Netherlands; ^3^Division of Health System Innovation and Research, Population Health Sciences, University of Utah, Salt Lake City, UT, United States; ^4^Department of General Internal Medicine, School of Medicine, University of Utah, Salt Lake City, UT, United States; ^5^Center for Health Promotion and Prevention Research, School of Public Health, University of Texas Health Science Center at Houston, Houston, TX, United States

**Keywords:** physical activity, implementation science, rehabilitation, musculoskeletal disorders, behavior change and communication

## Abstract

**Background:**

Coach2Move is a person-centered physical therapy intervention that has demonstrated success in changing physical activity behaviors among older adults in the Netherlands. In this manuscript, we describe how we developed an implementation plan for Coach2move in a U.S. population and healthcare system using Implementation Mapping.

**Methods:**

We established an implementation planning team of researchers, patients, and clinicians. The Consolidated Framework for Implementation Research provided an overall structure for consideration of the context for implementation. Implementation Mapping guided the planning process. The implementation planning team worked sequentially through the five tasks of Implementation Mapping (1) Identify needs, program adopters and implementers; (2) Identify adoption and implementation outcomes, performance objectives, determinants, and matrices of change; (3) Choose theoretical models and implementation strategies; (4) Produce implementation protocols; (5) Evaluate implementation outcomes. In this manuscript, we identify our evaluation plan but not results as data collection is ongoing.

**Results:**

Clinic managers and physical therapists were identified as program adopters and implementors. Performance objectives necessary steps to achieving implementation outcomes were linked to Coach2Move fidelity indicators with implementation by the physical therapist. These included delivery of person-centered care, motivational interviewing, meaningful goal setting, shared decision-making in planning, and systematic monitoring and follow-up. Determinants linked to these performance objectives included knowledge, outcome expectations, skills and self-efficacy, and perceived norms. Implementation strategies were selected based on a review of methods effective for influencing these determinants. This resulted in four primary strategies (1) educational meetings and dynamic training, (2) peer-assessment meetings, (3) changing the electronic health record template, and (4) reminders and prompts. Measures of intervention acceptability, appropriateness, and feasibility will be collected after training and early in implementation. Fidelity and effectiveness measures will be collected over the next 12-months.

**Conclusion:**

Implementation mapping provided a systematic process for identifying what physical therapists would need to implement Coach2Move with fidelity. The result was a matrix linking behavioral determinants and performance objectives. These matrices of change allowed for systematic identification and tailoring of implementation strategies to the needs of our population and setting. The process was acceptable to diverse stakeholders, facilitated communication across stakeholders.

## Introduction

Chronic musculoskeletal (MSK) conditions such as low back pain and osteoarthritis are a leading cause of years lived with disability globally (1). MSK conditions not only have a profound impact on function but are one of the most common reasons adults seek medical care (2). Clinical practice guidelines recommend physical activity (PA) as the cornerstone of disease management and many individuals are referred to physical therapy (3–5). While people with MSK report improved pain and function with increased PA (6–8), few successfully sustain PA after physical therapy and subsequently still struggle with symptom management (9–14). There is a critical need to develop and test implementation strategies that facilitate the delivery of evidence-based interventions to improve PA in the physical therapy setting.

Coach2Move is a physical therapist delivered intervention shown to increase PA after physical therapy in community-dwelling older adults (15). In collaboration with Coach2Move researchers, we adapted the intervention to a U.S. population of middle age and older adults with chronic MSK conditions. The aim of the current project was to identify implementation strategies appropriate for our clinical environment. Implementation mapping provided a systematic process, using five main tasks, for selecting and planning our implementation strategies (16). This process was developed based on the intervention mapping framework and uses community stakeholder input, behavioral and implementation theories, and empirical findings to guide the output (17).

The Consolidated Framework for Implementation Research (CFIR) and social cognitive theory guided our consideration of the context and individual determinants of change (18–20). The CFIR domains and menu of constructs provided a practical guide to assessing a range of potential barriers and facilitators to implementation in our environment. Social cognitive theory posits that cognitive, behavioral, and environmental factors influence behavior change and is often applied at an individual level (21). These factors interact and support a central premise that individuals strive for a sense of agency. Both the CFIR and social cognitive theory highlight the need to consider the environment in which a behavior occurs and the interaction how an individual interacts with an intervention to influence implementation.

Coach2Move is a paradigm shift in the physical therapist's communication from a traditional approach of the physical therapist as expert to one which includes patient expertise. Despite known effectiveness of person-centered care, implementation in physical therapy has been challenging (22, 23). Physical therapists lack self-efficacy and skills in communication around sensitive topics such as mental health and emotional distress (23, 24). They also find it difficult to elicit motivation, address ambivalence, and partner with patients on strategies that change PA in everyday life (25). In Coach2Move, physical therapists train in motivational interviewing to engage patients in identifying meaningful goals, monitor progress, and plan for self-management through sustainable changes in PA (26). Coach2Move has demonstrated acceptability with patients and physical therapists, effectiveness in sustaining PA beyond an episode of physical therapy care, and cost-effectiveness (15, 27, 28). Differences between core components of Coach2Move and routine physical therapy are highlighted in Table 1. These core components were the essential structure for our performance outcomes within the Implementation Mapping process.

**Table 1:** Comparison of Coach2Move core components and routine physical therapy.

**Routine physical therapy**	**Coach2Move physical therapy**	**Performance objectives**
Diagnosis centered: focus on common conditions specific impairments	Person centered: focus on meaningful activities at home with help from social network	Tailors program to individual functional needs and readiness to change
Gathers information primarily through closed-ended questions, “provider-centric”	Gathers information using open-ended questions, reflections, and summaries	Uses motivational interviewing to elicit reasons to change physical activity
Goals often set by physical therapist	Shared decision-making about meaningful treatment goals	Identifies inspiring and measurable goals
Focused on impairment and short-term management of symptoms	Planning for long-term solutions to chronic symptoms management	Explicit conversation on physical activity and the relationship of physical activity and the MSK condition
Physical therapist directs plan (“Physical therapist as expert”)	Physical therapist supports self-management and empowerment with negotiated planning (Identifies “Patient as expert” in their life)	Empowers patient to monitor their own progress and identify solutions
Varied application of standardized performance tests and patient-reported outcomes. Primarily performed at baseline.	Systematic monitoring using patient reported outcomes and performance measures throughout follow-up and discussed with patient.	Uses appropriate measurement to discuss progress across the episode of care

The goal of Coach2Move is to equip physical therapists with the tools to successfully promote PA behavior change in patients with chronic MSK conditions. This manuscript describes our approach to the development of a multifaceted implementation strategy, using Implementation Mapping, to facilitated delivery of Coach2Move in a U.S. health system.

## Methods

### Setting

This study was conducted within and academic health system, University of Utah Health (UHealth). We considered all 7 outpatient physical therapy clinics located in the greater Salt Lake City area and Park City in our implementation planning. These clinics represent 122 physical therapists and 2 different management structures.

### Target Participants

Coach2Move will target patients who are 50 years and older with a chronic MSK condition (i.e., chronic low back pain, hip or knee osteoarthritis) and receiving outpatient physical therapy. Physical therapists will be eligible to participate if they work more within UHealth, are scheduled more than 19 h/week, and routinely treat middle-age and older adults with chronic MSK conditions (>30% of average workload).

### Implementation Planning

We established a diverse implementation planning group to design the multifaceted implementation strategy. This group consisted of researchers, patient stakeholders, physical therapists, social workers with expertise in motivational interviewing, and Coach2Move developers. Patient and physical therapist stakeholders were recruited from UHealth. Patient stakeholders were 50 years or older and had a chronic MSK condition for which they had received physical therapy. Patient stakeholders had participated previously in participatory research. Physical therapist stakeholders were selected to represent clinics with differing management structures and routinely manage middle age and older adults with chronic MSK conditions. Researchers at the University of Utah guided the process and were the primary point of contact with each stakeholder group.

### Logic Model

**Figure 1:**
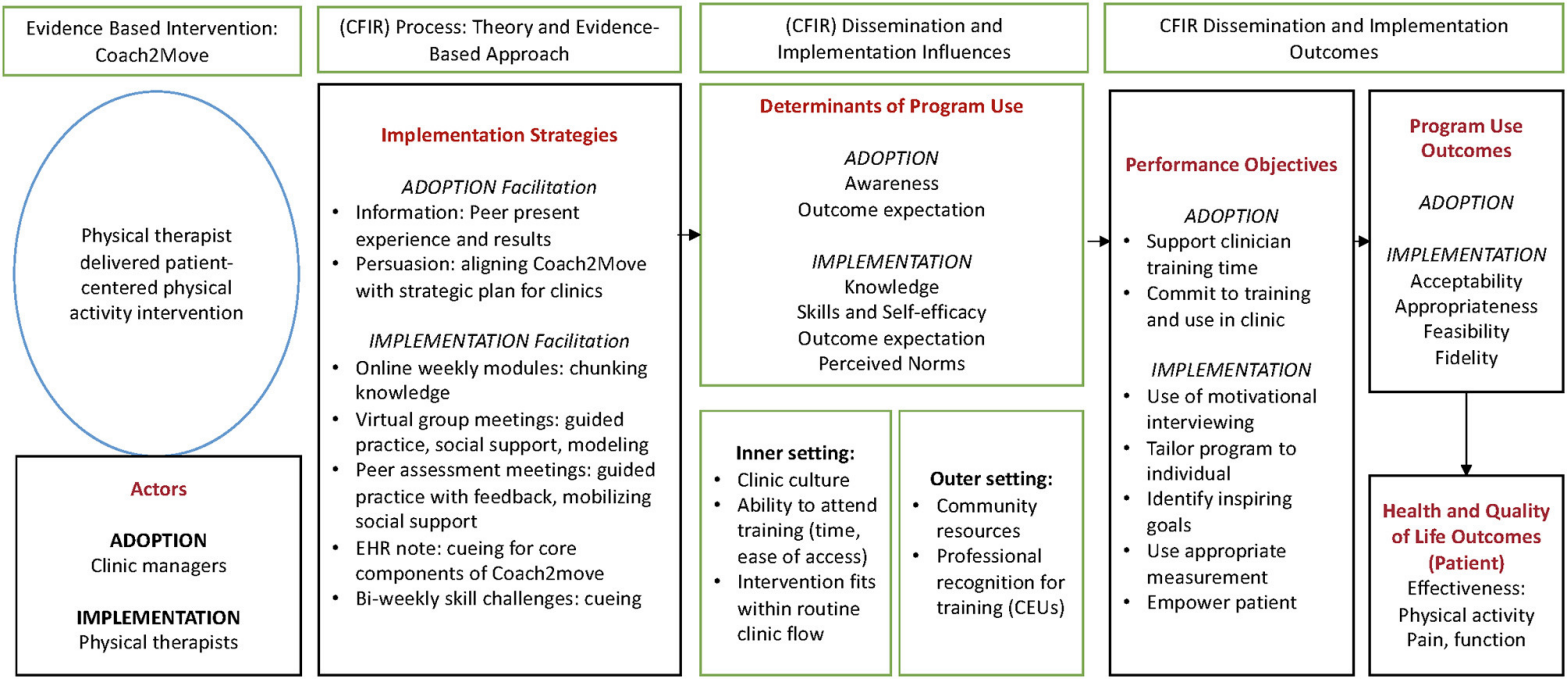
Implementation logic model for Coach2Move guided by intervention mapping and the Consolidated Framework for Implementation Research (CFIR).

The planning group first reviewed the outline of implementation strategies used previously by Coach2Move researchers. From this foundation, we used the Implementation Mapping process and the Consolidated Framework for Implementation Research (CFIR) to consider constructs and domains likely to influence implementation within our setting. CFIR helped us identify potential contextual factors that could influence implementation both in the current study and with future implementation. We worked sequentially through each Implementation Mapping task. Throughout the process, we reviewed behavior change models and literature to help prioritize determinants of change and implementation strategies most likely to be effective. An overview of the logic model is provided in Figure 1.

### Implementation Mapping Tasks

Implementation Mapping starts with an implementation needs assessment and identifying program adopters, implementers, and maintainers (Task 1). Given our early stage of implementation, we focused on adoption and implementation. We identified adoption and implementation needs through structured and unstructured interviews of physical therapist and clinic managers. In Task 2, we created a logic model for determining how our implementation strategies would effect change. We started with identifying adoption and implementation outcomes. We then identified the performance objectives necessary to achieve our adoption and implementation outcomes and deliver the core components of Coach2Move (Table 2). Our final product of Task 2 was a matrix of performance objectives with determinants of change. This matrix identified what needed to be changed through the implementation strategy to influence performance objectives and subsequently achieve adoption and implementation outcomes. In addition, this matrix provided a structure for considering how we would evaluate change over the course of implementation. In Task 3, we matched the matrices of change with implementation strategies. With an understanding of the behavioral determinants to target, the context, and selected strategies, we produced the implementation protocol and materials (Task 4). Finally, we established a plan for evaluating implementation outcomes (Task 5) which included establishing methods for measuring implementation outcomes and process determinants. Implementation outcomes collection is ongoing and will not be reported here.

**Table 2:** Implementation needs assessment, adopter, implementer, and maintainers.

**Role**	**Immediate**	**Future**
Adopters	**Clinic managers** *Rationale:* Advocate/Supports the importance of the program Support for training time Approval for change in documentation templates **Physical therapists** *Rationale:* Decision to actively participate in training	**Heath systems** *Rationale:* Increase visibility of program, adapt environment, support maintenance and monitoring **Referring Providers** *Rationale:* Increase acceptability with patients **Payers** *Rationale:* Potential to change payment structure
Implementers	**Physical Therapists** *Rationale:* Core components of Coach2Move require physical therapist expertise alongside person-centered communication	**Physical therapy assistants** *Rationale:* Assistants assume a portion of patient care visits and can improve continuity of coaching toward goals
Maintainers	**Coach2Move clinician leaders** *Rationale:* Provides for professional development and leadership opportunities while supporting clinic processes	**Coach2Move network of clinicians** *Rationale:* Social network supports communication across settings and provides opportunity to examine adaptation needs

The planning team acknowledged that successful delivery of person-centered care is dependent on the health care system, external context, clinicians, and interactions between these components (29). In this project, we selected to focus primarily on determinants associated with individual clinicians, specifically the physical therapist. Analysis of implementation outcomes will include both clinician and patient level data. This project was approved by the Institutional Review Board (ID 00109256) at the University of Utah and all participants were included only after providing informed consent.

## Results

### Task 1: Conduct a Needs and Assets Assessment and Identify Program Adopters, Implementers, and Maintainers

In prior work, we identified strengths and limitations of routine physical therapy in supporting patients with chronic MSK conditions to sustain PA (30). Briefly, physical therapists strongly identified with their role in promoting PA and reported a desire to develop strategies for patients who were less engaged or ambivalent about behavior change. Physical therapists reported difficulty eliciting motivation and empowering patients with strategies for continued PA beyond the clinical episode.

The stakeholder group reviewed these assets and needs alongside the components and characteristics of the Coach2Move intervention. This step focused on identifying the actors for adoption, implementation, and maintenance (16). Discussions incorporated the need for adaptations based on clinical time constraints, training time and associated costs to the clinic, development of training materials, and incorporation of future cohorts. For example, stakeholders raised the question about using other clinical staff such as a physical therapy assistant or health coach to facilitate the behavioral change component and reduce the time demand on the physical therapist. Based on review of data from the original Coach2Move implementation, it was determined that the behavior change intervention was more effective when integrated into the clinical decisions about treatment.

Given the stage of the research, we also decided to focus on immediate adoption and implementation needs but identified considerations for future adoption, implementation, and maintenance. Results of Task 1 are summarized in Table 2.

### Task 2: Identify Adoption and Implementation Outcomes, Performance Objectives, Determinants, and Create Matrices of Change

Working through Task 2, the implementation planning group discussed what actions would lead to successful implementation of Coach2Move. Adoption was focused on the clinic managers and physical therapists (Table 3) and considered the inner and outer context. Meetings with clinic managers outlined the training proposal and aims of Coach2Move highlighting benefits to physical therapists and patients. We reviewed the managers' needs and considered how they aligned with Coach2Move. Managers expressed a critical need to improve availability for new patient visits. We highlighted how Coach2Move was expected to reduce the overall number of physical therapy visits. By reducing the number of return visits, the schedule would have more availability for new patients. The managers also requested efforts to minimize the impact of scheduled training on clinic productivity. To accommodate these requests, we staggered training cohorts and scheduled peer assessment meetings at two different times of the day. Through these discussions and negotiations, we were able to garner management support to meet adoption performance objectives.

**Table 3:** Implementation outcomes and performance objectives.

**Target/role**	**Adoption and implementation outcomes**	**Performance objectives**
Clinic manager *adopter*	Manager supports training of clinicians in Coach2Move	•Agrees to participate in Coach2Move and promotes with clinicians •Allows for 50% of training time to be schedule from normal clinic hours for continuing education credits
Physical therapist *adopter*	Physical therapist acknowledges training commitment and agrees to participate	•Completes 80% of training activities •Uses Coach2Move documentation template
Physical therapist *implementer*	Physical therapist incorporates Coach2Move core components with eligible patient interactions with >70% fidelity	PT addresses each core component: •Focused conversations on physical activity •Uses motivational interviewing to elicit reasons to change PA •Tailors program to individual functional needs and readiness to change •Identifies inspiring and measurable goals •Uses appropriate measurement to discuss progress across the episode of care •Empowers patient to monitor their own progress and identify solutions
Physical therapist *implementer*	Physical therapists reflect and improve on their implementation of Coach2Move core components	•PTs use peers to support in problem solving •PTs identify missing information/skills and redundancies that could be addressed to improve acceptability

Physical therapists were invited to participate if they worked routinely with older adults who had chronic MSK conditions. To influence adoption, we obtained accreditation for the training program from our state physical therapy association. This allowed clinicians to schedule education time rather than personal time to participate, which was preferred by both physical therapists and clinic managers. Performance objectives for physical therapist adoption included a commitment to participate in training and to use the training in clinical care.

Implementation performance objectives were structured around the core components of Coach2Move (Tables 1, 3). Using a list of quality indicators associated with positive Coach2Move outcomes (28), we outlined sub-behaviors a physical therapist would need to exhibit to implement Coach2Move with fidelity.

Next, we specified determinants for adoption and implementation. Researchers at University of Utah Health performed a literature review identifying factors associated with clinician delivery of behavioral interventions (13, 24–27). We met with Coach2Move developers to identify prior implementation experiences and contrasted this with the literature review. With Social Cognitive Theory as an underlying structure, we presented proposed determinants to physical therapist stakeholders and social work partners for feedback (19, 31). The planning group prioritized determinants based on their strength of association with the performance outcome and their changeability. Primary determinants identified for delivering Coach2Move core components were knowledge, skills and self-efficacy, outcomes expectations, and perceived norms. These determinants were considered fundamental and have been shown to be associated with healthcare provider behavior (19). From these determinants we created matrices of change objectives. Table 4 demonstrates a sample of the matrix used for implementation performance objectives. These objectives were formulated by assessing what factors needed to be present to achieve the performance objective and why a physical therapist might change their behavior to meet the performance objective. Creating this matrix provided a foundation for selecting implementation strategies. Consider the performance objective “*Uses appropriate measurement across the episode of care”* as an example of how to use this matrix. Essential to using measurement tools is having knowledge of the tool and how to interpret the results. Skills and self-efficacy are needed to enable discussions of these results with patients. Implementation strategies to address these determinants may include instruction or lecture, simulation, and feedback. Motivation to routinely use systematic measurement is also dependent on what a physical therapist can gain (outcome expectations) and what they believe is expected of them (perceived norms). Implementation strategies were then selected based on their ability to affect the determinant, such as using testimonials to influence outcome expectations or peer-assessment to change perceived norms.

**Table 4:** Matrices of change objectives for implementation of Coach2Move by physical therapists.

**Performance objectives**	**Determinants**			
	**Knowledge**	**Skills and self-efficacy**	**Outcome expectations**	**Perceived norms**
Evaluate personal strengths and challenges in delivering Coach2Move	Describe components of effective delivery strategies alongside self-evaluation	Expresses confidence reflecting on and assessing own practice	Expects reflective practice will improve proficiency	Recognizes responsibility for own professional development
Use motivational interviewing to elicit reasons to change PA	Describe key components of motivational interviewing	Demonstrate proficiency in motivational interviewing skills Take action, e.g., use Coach2Move template to guide conversations about PA	Expect that motivational discussions around physical activity will increase patient activation and engagement	Recognizes that motivational interviewing is within the scope of physical therapy practice and aligns with the vision of the profession.
Tailor program to individual functional needs and readiness to change	Describe potential analyses for common functional impairments Explain how to modify treatment to align with patient presentation	Design task analysis appropriate for patient goals Confident in adapting treatment plan to patient presentation	Evaluate how task analysis can improve patient engagement, efficiency, and treatment planning	
Identify inspiring and measurable goals	Describe how to identify and quantify an inspirational goal	Demonstrate how to progress from a functional impairment to understanding a patient's motivation to change	Describe how an inspirational goal improves patient adherence	
Use appropriate measurement across the episode of care	Select appropriate measurement tools for patient presentation	Explain how measurement relates to patient goals	Expect that regular measurement can improve decision-making	Recognize professional obligation to support clinical decisions through measurement
Empower patient to monitor their own progress and identify solutions	Identify different methods for negotiating a treatment plan with patient	Demonstrate ability to collaborate with patient on treatment planning Demonstrate MI techniques to elicit patient ideas and commitment to monitoring	Recognize that empowering patients will lead to improved adherence at the patient level and job satisfaction for the physical therapist	Recognize physical therapists need to improve person-centered communication to increase engagement and self-management

### Task 3: Choose Theoretical Models; Select or Create Implementation Strategies

For this task, we again reviewed the literature to identify effective implementation strategies for changing clinician behaviors. Continuing education courses are a common method for physical therapists to acquire new knowledge. These courses, whether in person or through e-Learning have a modest effect on changing clinician behaviors that wanes over time (32, 33). Training components that improve implementation include multiple exposures, interactivity, longer training periods, and focusing on outcomes important to clinicians (33, 34). Specific to physical therapy, reflection, simulations, self- and peer-assessment improve self-efficacy and commitment to behavior change (35–37). Deliberate practice and structured feedback facilitates changes in person-centered communication (38). In summary, components identified with successful change in clinician practice include shaping knowledge, feedback and monitoring, social support, and social comparison (39). Using this summary, our prioritized list of determinants, select theories, and prior Coach2Move experience, we identified practical applications for addressing each determinant.

For an example, consider the performance objective presented in Table 4, “*Empower a patient to monitor their own progress and identify solutions*.” An associated change objective was “*Demonstrate the ability to collaborate with patients on treatment planning*.” To meet this change objective, physical therapists need skills and self-efficacy in communication strategies that support collaborative treatment planning (40). Active learning strategies that include practice, review, and repetition are effective methods for improving skills and self-efficacy (41–43). Having outlined this, we knew we needed to operationalize modeling, guided practice, and feedback in Task 4.

Practical applications were cross-referenced with strategies as outlined by Expert Recommendations for Implementing Change (44). The end results was our multifaceted implementation plan (Table 5). Our strategy for adoption by the clinic manager was to develop a partnership and adapt the training approach to minimize disruption of patient care. Strategies aimed at adoption by physical therapists included incentives (continuing education time) and allowance structure (protected training time) and the identification of early adopters. Implementation strategies informed by Task 3 included: (a) educational meetings and dynamic training, (b) organizing three clinical implementation team meetings in which clinicians reviewed challenges of implementation with discussions of potential solutions and provided self- and peer- assessment of skills, (c) modifying the electronic health record system to include a Coach2Move template prompting the use of skills acquired in training and reflection on practice, and (d) reminding clinicians using bi-weekly emails reviewing information from training and provide clinical examples or prompts. Of note, physical therapists found the peer assessment meetings to be particularly helpful and motivating. They recommended scheduling more of these meetings over time for peer support and problem solving, prompting us to consider creating a learning collaborative as an opportunity to sustain the Coach2Move intervention.

**Table 5:** Coach2Move (C2M) implementation intervention plan.

**Stage**	**Determinants/change objectives**	**Theoretical change methods**	**Practical applications**
Adoption Agent: Clinic Manager	Awareness Perception of C2M Outcome Expectations	Information Persuasion Role modeling	C2M presentations from Dutch colleagues Decisional balance handout on adoption of C2M
Adoption: Agent: Physical therapist	Awareness Perception of C2M Outcome Expectations	Persuasion Communication Mobilization	Email invitation to participate (template) Accredit training through professional organization to provide continuing education units C2M presentations from Dutch colleagues
Implementation Agent: Physical therapist	Knowledge Skills and self-efficacy Outcome expectation Normative beliefs Social influence	Chunking Modeling Guided practice with feedback Role-modeling Persuasion Cue altering Mobilizing social support	Core components in 6 modules completed weekly Virtual meetings for problem solving and guided practice Peer reports of positive outcomes Peer-assessment: skills practice and problem solving C2M specific charting template
Maintenance Agent: Clinician leader Clinic managers	Outcome expectations Skills and self-efficacy Feedback and Reinforcement	Information Persuasion Technical assistance	Face to face meetings to discuss maintaining Continued access to online training materials Public recognition of clinician leaders Promote use of clinic leaders for problem solving Continued managerial support Add-in modules recommended by participants

We did not constrain participation to sites where the entire clinical site chose to participate. Instead, we described the study to physical therapists across six clinics in a metropolitan region and invited them to participate leveraging early adopters (19, 45, 46). Of 82 physical therapists, 32 (39%) participated and were considered to represent innovators and early adopters. We considered this an advantage for our stage in development as these individuals could further shape the intervention through critical review of implementation components and stand out as opinion leaders (47).

### Task 4: Produce Implementation Protocols

In Task 4 the planning group moved to designing the program components and materials. Prior Coach2Move implementation included a 2-day in-person training to address knowledge, skills, and self-efficacy. This is common practice for professional continuing education and has demonstrated prior effectiveness (15, 28). We were unable to adopt this method for two reasons: (1) COVID-19 restrictions, and (2) the clinic manager's request to limit the impact on clinical scheduling which did not allow for clinicians to schedule training time all on the same day. We altered training to provide asynchronous and synchronous learning. Online training modules were developed and delivered through a web-based learning management system, (Canvas, Instructure Inc, SLC, UT). We created 6 weekly modules of approximately 1-h covering the 6 core components of Coach2Move. Each module included interactive elements such as challenges for clinical application and discussion boards. Modules included knowledge dissemination, modeling the behavior using clinical examples, and an example of a Coach2Move trained physical therapist with a standardized patient. The online training was supplemented with two 2.5-h virtual meetings. This allowed time to discuss challenges, questions, and hear about peer successes. These meetings also used modeling, guided practice, and feedback for further skill development.

**Figure 2:**
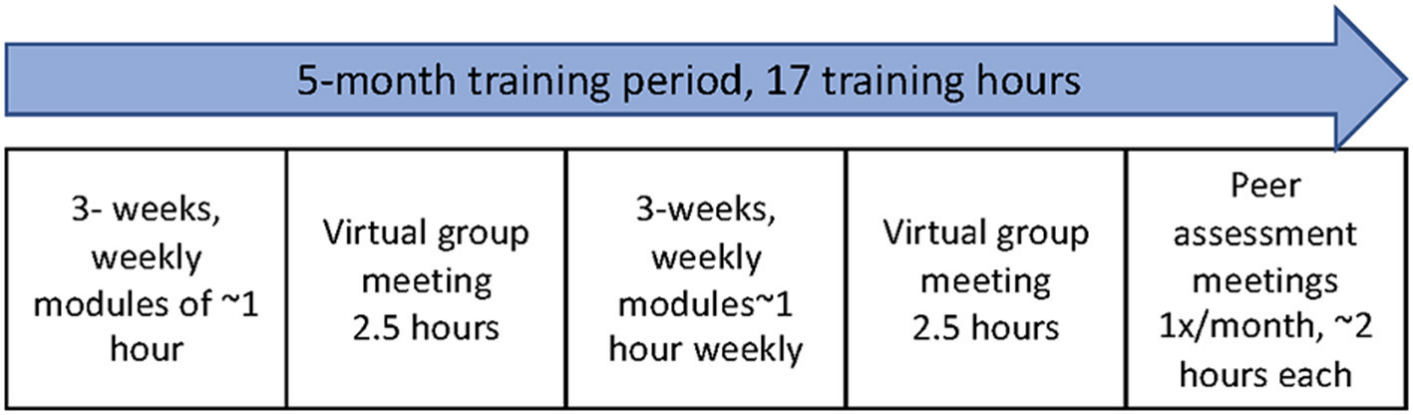
Implementation timeline and dose.

Peer-assessment meetings were held once monthly over 3-months for skills practice, feedback, and social influence. In preparation, we developed 2 common clinical scenarios, trained a standardized patient, and created feedback forms aligned with quality indicators for Coach2Move. Each physical therapist recorded an interview intake with the standardized patient. In addition, physical therapists recorded a clinic encounter with a patient appropriate for Coach2Move. Using the recorded videos and feedback forms, physical therapists partnered with a peer for guided self-assessment and a peer- assessment. This provided opportunities to provide affirmations and discuss alternate strategies. Physical therapists were provided a Coach2Move chart template (integrated into the electronic health record) and bi-weekly email reminders to support clinical integration through cueing. Figure 2 provides an overview of temporality and dose of our implementation strategies.

### Task 5: Evaluate Implementation Outcomes

**Figure 3:**
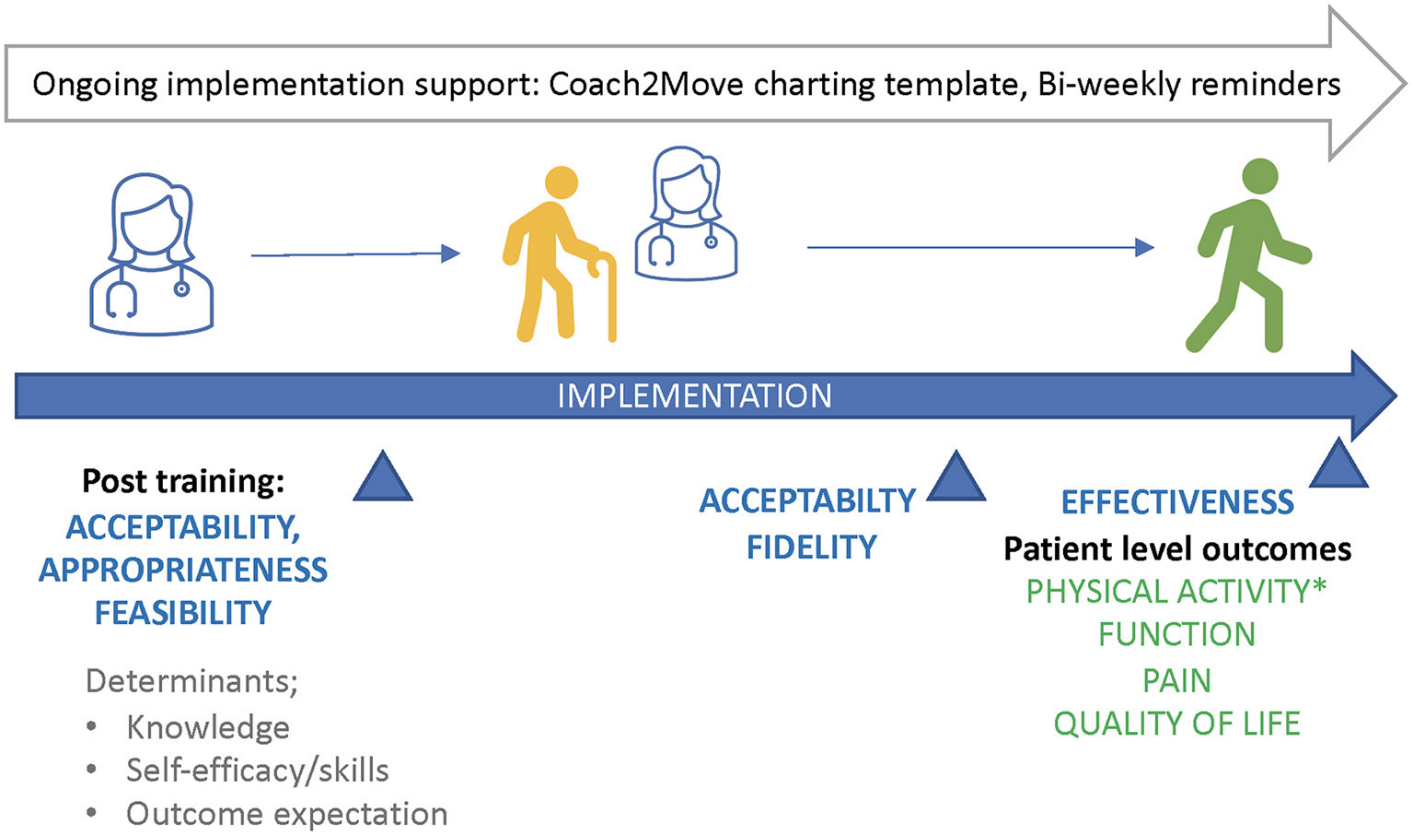
Implementation outcomes and timeline for collection.

Our final task was planning evaluation of implementation. We planned outcome assessments at both the physical therapist level and patient level and across several different time points (Figure 3). We considered outcomes appropriate to the early phase of implementation (48, 49). Primary outcomes of interest included acceptability, appropriateness, feasibility, fidelity, and effectiveness. We surveyed physical therapists on the acceptability, appropriateness, and feasibility of Coach2Move using the Acceptability of Intervention, Intervention Appropriateness, and Feasibility of Intervention measures (12). Each measure has four items relevant to the concept of interest and 5-response options ranging from “*completely disagree*” to “*completely agree*.” For example, the Feasibility of Intervention asks the physical therapist to score their agreement with the statement, “*Coach2Move seems doable*.” A qualitative assessment of clinician and patient experience with Coach2Move after 6-months of implementation will further examine acceptability, appropriateness, and feasibility.

We also developed measures to understand the impact of our implementation on the determinants identified in our mapping process. For knowledge and self-efficacy, we created surveys to capture physical therapist beliefs and confidence in delivering Coach2Move. To measure skills, we created an observational coding tool to score physical therapists conducting an interview with a simulated patient across two different scenarios. The coding tool was developed using quality indicators from the original Coach2Move implementation and input from our social work and physical therapist stakeholders.

Coach2Move fidelity indicators previously developed for Coach2Move implementation in the Netherlands was added to the physical therapy documentation template (28). The template provides cueing for the core elements of Coach2Move. Effectiveness will be measured at the patient-level through self-reported PA and objective measures of PA using a commercially available activity monitor. Planned analyses include the increase in PA at 6-months with the Coach2Move intervention and the association between fidelity and effectiveness. Proximal outcomes of the training have been collected and are being analyzed while additional implementation outcomes are ongoing with an expectation for completion in January 2023.

## Discussion

Person-centered care is a critical component in improving health behaviors and clinical outcomes in patients with chronic MSK conditions (15, 50, 51). Successful delivery requires understanding the patient as a whole and adapting to the patient's disease experience (35). Physical therapists acknowledge the need for a person-centered approach to care but continue to have difficulty implementing many components of person-centered care (22). The patient-physical therapist interaction is often characterized as practitioner dominant with physical therapists finding it challenging to balance their own agendas with that of the patient (52, 53). Coach2Move is an evidence-based intervention for physical therapists in which person-centered care is foundational and improves clinical outcomes for patients. In this study, implementation mapping allowed our team to identify determinants of change and develop a comprehensive implementation plan that would facilitate uptake of Coach2Move.

Implementation focused on the questions, “*Why would clinic managers adopt Coach2Move?*,” “*What do physical therapists need to implement Coach2Move?”* and “*Why is person-centered care difficult?”* Changing communication practice to elicit motivation and empower patients with self-advocacy requires new skills and patterns of practice for most physical therapists (25, 28). Person-centered care with a focus on behavior change has been described as “learning a new language” and requires restructuring of the consultation framework (25). Working through the implementation mapping process within the CFIR framework, we identified individual level determinants for change and the interplay between the context and actors. Knowledge, skills and self-efficacy, outcomes expectation, and perceived norms were identified as determinants to influence. These were the targets of the implementation strategies which included educational meetings, implementation team meetings, practice, and feedback. Context interventions including creating social support and using prompts withing the electronic health record.

Explicitly identifying matrices of change allowed us to integrate and discuss behavior change models and identify intended proximal outcomes of our implementation strategy (54). Proximal outcomes allow us to better understand how our implementation strategies may be affecting change. For example, we hypothesized training would immediately improve motivational interviewing skills and that delivery of Coach2Move was dependent on proficiency in motivational interviewing. By assessing these skills pre- and post-training, we will understand the immediate impact of training. Through fidelity measures over the course of study enrollment, we will understand the relationship between motivational interviewing skill and Coach2Move delivery. If physical therapists demonstrate proficiency in motivational interviewing but fail to apply this skill in the clinic, we have evidence of the need to examine other determinants influencing implementation. The change matrices also highlighted the need to affect multiple determinants with our implementation strategies. Multifaceted strategies to change physical therapist behaviors have shown greater effect but their use remains limited with a strong dependency on educational meetings and reminders (55).

The planning group found implementation mapping to be particularly helpful in three ways (1) organizing discussions and input across stakeholders, (2) identifying how an implementation strategy would affect change, and (3) creating a broad overview of the body of research. Using the logic model presented in Figure 1, all stakeholders had an overview of the intent and essential task of the mapping process. Each task helped to complete the logic model and was suitable for stakeholders of different expertise. It was difficult to schedule planning meetings with all stakeholders at the same time. Having the logic model and each implementation mapping task as a working document allowed us to get feedback from each stakeholder group without requiring a full planning group meeting.

The logic model and specificity of each task allowed the planning group to create a broad overview of research gaps and identify the specific purpose of this study. This prompted discussion about our stage of implementation research (early) (48) and influenced our focus. It also allowed for discussions about how moderators we leveraged in the current study might need to be addressed differently in the future. As noted, physical therapists self-selected to participate. This represents a sample of individuals motivated to adopt and implement the training (46). Training across a broader population may require alternate strategies to address both moderators and mediators. Using the CFIR framework also prompted additional questions about the influence of the outer structure, inner structure, and individual actors. The framework allowed us to record these considerations to be addressed in future implementation efforts.

## Conclusion

Through the process of Implementation Mapping, our multidisciplinary stakeholder group produced a comprehensive training program to implement Coach2Move, a physical therapist delivered PA intervention for patients with chronic MSK conditions. Many healthcare providers recommend PA, but there is often little structured support for behavior change. Training physical therapists to effectively support patients in PA behaviors fills a much-needed gap and has the potential to significantly reduce the burden of chronic MSK conditions for both individuals and health systems. This study highlights a systematic approach for selecting implementation strategies to implement Coach2Move by considering how these strategies are expected to affect change. This study also highlights how Implementation Mapping can be used as a working document to integrate input from multiple stakeholders. Results of Coach2Move implementation will be reported at a future date.

## Data Availability Statement

The original contributions presented in the study are included in the article/supplementary material, further inquiries can be directed to the corresponding author/s.

## Ethics Statement

The studies involving human participants were reviewed and approved by University of Utah Institutional Review Board. The patients/participants provided their written informed consent to participate in this study.

## Author Contributions

AT, RH, JF, TH, MN-vD, MC, and MF contributed to conception and design of the study. AT, JW, MN-vD, and TH organized and gathered stakeholder feedback. AT wrote the first draft of the manuscript. All authors contributed to manuscript revisions, read, and approved the submitted version.

## Funding

This project was funded under contract/grant number 1K01HS026518 from the Agency for Healthcare Research and Quality (AHRQ), U.S. Department of Health and Human Services.

## Author Disclaimer

The opinions expressed in this article are those of the authors and do not reflect the official opinion of AHRQ or the U.S. Department of Health and Human Services.

## Conflict of Interest

The authors declare that the research was conducted in the absence of any commercial or financial relationships that could be construed as a potential conflict of interest.

## Publisher's Note

All claims expressed in this article are solely those of the authors and do not necessarily represent those of their affiliated organizations, or those of the publisher, the editors and the reviewers. Any product that may be evaluated in this article, or claim that may be made by its manufacturer, is not guaranteed or endorsed by the publisher.
